# Nitrogen fertilization and CO_2_ concentration synergistically affect the growth and protein content of* Agropyron mongolicum*

**DOI:** 10.7717/peerj.14273

**Published:** 2022-10-31

**Authors:** Aiyun Xu, Lihua Zhang, Xiaojia Wang, Bing Cao

**Affiliations:** School of Agriculture, Ningxia University, Yinchuan, China

**Keywords:** Elevated CO2, N-fertigation, Leaf nitrogen content, Root uptake, Nitrate assimilation

## Abstract

**Background:**

The nitrogen (N) and protein concentrations in plant tissues exposed to elevated CO_2_ (eCO_2_) generally decline , such declines in forage grass composition are expected to have negative implications for the nutritional and economic value of grass. Plants require N for the production of a photosynthetically active canopy and storage proteins in the tissues, whose functionality will strongly influence productivity and quality. The objective of this study was to investigate whether eCO_2_ plus N-fertilization increases growth and N nutrition of *Agropyron mongolicum*, and the dependence of this improvement on the coordination between root and leaf development.

**Methods:**

We analyzed *A. mongolicum* from field-grown within the open-top chambers (OTCs) facility under two atmospheric CO_2_ (ambient, 400 ± 20 µmol mol^−1^, aCO_2_, and elevated, 800 ± 20 µmol mol^−1^, eCO_2_) and three N-fertigation treatments (control, low N-fertigation , and high N-fertigation) for two months.

**Results:**

Elevated CO_2_ plus N-fertigation strongly increased shoot and root biomass, and the nitrogen and protein concentrations of *A. mongolicum* compared to those plants at aCO_2_ levels. Increased N content in leaves and reduced specific leaf area (SLA) at a high N supply could alleviate photosynthetic acclimation to eCO_2_ and drive the production of greater shoot biomass with the potential for higher photosynthesis, productivity, and nutritional quality. The increased root length (RL), the ratio of total aboveground N taken up per RL (TN/RL), stomatal conductance (Gs), and transpiration rate (Tr) contribute to the transpiration-driven mass flow of N, consequently increasing N uptake by roots. In addition, a smaller percentage of N remained as unassimilated nitrate (${\mathrm{NO}}_{3}^{-}$) under eCO_2_, indicating that assimilation of ${\mathrm{NO}}_{3}^{-}$ into proteins was not inhibited by eCO_2_. These findings imply that grass productivity and quality will enhance under anticipated elevated CO_2_ concentration when effective management measures of N-fertilization are employed.

## Introduction

Atmospheric carbon dioxide (CO_2_) concentration has risen from 270 µmol mol^−1^ before the industrial revolution to 414 µmol mol^−1^ in 2020 (Domiciano et al., 2020), and the rate of increase is predicted to hasten during this century (IPCC, 2013). Elevated CO_2_ concentrations (eCO_2_) have a significant impact on plant growth, productivity, and quality in natural and agricultural systems (Burgess & Huang, 2014; Liu, Tian & Zhang, 2016). It is well established that plants can benefit from the augmented atmospheric CO_2_ concentration through the “CO_2_ fertilization effect”, particularly for C_3_ species (Burgess & Huang, 2014). Since C_3_ plants use the carboxylase enzyme RuBisCO (ribulose-1,5-bisphosphate carboxylase-oxygenase) to fix CO_2_ from the air and obtain 3-carbon intermediate molecules as the first step in photosynthesis, lose a portion of their fixed CO_2_ to oxidative photorespiration under present CO_2_:O_2_ ratios because RuBisCO is also an oxygenase (Ainsworth & Long, 2005; Reich et al., 2018). By contrast, in C_4_ plants, a different enzyme (phosphoenolpyruvate carboxylase) with a high affinity for CO_2_ and lacking oxygenase activity first incorporate CO_2_ into a 4-carbon intermediate, which is then shuttled to specialized bundle sheath cells where CO_2_ isreleased, and a high CO_2_:O_2_ ratio results in a lower rate of photorespiration. Thus, C_3_ plants exhibit increased photosynthesis as increasing CO_2_:O_2_ ratios reduce rates of photorespiration and increase rates of carboxylation, while the photosynthetic rate of C_4_ plants is hardly affected by eCO2 levels in the air (Reich et al., 2018). Meanwhile, the eCO_2_ can decrease stomatal conductance (Gs) and transpiration rate (Tr) thereby increasing water use efficiency (Li, Wang & Liu, 2021), which can mitigate the negative climate effects accompanied by rising atmospheric CO_2_ effects, such as global warming and precipitation pattern (Zheng et al., 2020). However, higher growth rates and substantial biomass accumulation dilute nutrients within their tissues. It has been reported that the nitrogen and protein concentrations in plant tissues exposed to eCO_2_ generally declined due to constrain by N availability.

Previous studies have shown that the eCO_2_ can decrease nitrogen (N) concentration in plant tissues, particularly under N-limited conditions. One reason for the lowered N concentration of plants grown under eCO_2_ is the dilution of N by extra carbohydrate accumulation (Li, Wang & Liu, 2021). On the other hand, the uptake of N through transpiration-driven mass flow depends on the Tr and N ion concentration in the rhizosphere (Plhák, 2003). The eCO_2_ could cause a decrease in N uptake rate per unit mass or length of root due to decreased Tr thereby decreasing the mass flow of ${\mathrm{NO}}_{3}^{-}$ from the soil to the root (Li, Wang & Liu, 2021; Mcdonald, Erickson & Kruger, 2002). For example, Bloom (2015) observed that lessened leaf protein content under eCO_2_ is linked with limitations in ${\mathrm{NO}}_{3}^{-}$ assimilation caused by the reduction in transpiration. Also, eCO_2_ induced N-deficiency and sink:source imbalance, especially in leaf scale, can aggravate another phenomenon that is commonly observed: photosynthetic acclimation to eCO_2_ (Halpern et al., 2018). CO_2_ acclimation is intrinsically related to a reduction of Rubisco, and a decrease in leaf gas exchange and carboxylation capacity (Erice et al., 2014), consequently resulting in decreasing carbon assimilation rates and limiting plant growth (Cohen et al., 2019; Zheng et al., 2019). Similarly, it has been previously described in meta-analyses that the reduction of leaf N content in plants responding to eCO_2_ directly affects protein content (Loladze, 2014) and thus affects carbon fixation. The reduction of photosynthetic weakens, though generally does not eliminate, the expected stimulation of eCO_2_ on growth (Jauregui et al., 2015).

Nitrogen (N) is the mineral element that plants require in the largest quantities (Fernando et al., 2017), and it is a key component of amino and nucleic acids (Cohen et al., 2019). Plants mainly acquire N from the soil as nitrate (${\mathrm{NO}}_{3}^{-}$ ) and ammonium (${\mathrm{NH}}_{4}^{+}$). Thus, fertilization with N is vital for plant growth and development. N-fertilization has been described to increase root growth, alter the shoot-to-root ratio, favor fine-root proliferation and modify root architecture of *Arabidopsis* plants (Jauregui et al., 2016). Such effects highlight the fact that roots play a central role in nutrient uptake and assimilation (BassiriRad, Gutschick & Lussenhop, 2001). In addition to the central role of the root, the increase in leaf N has also been suggested. As leaf N directly affects photosynthetic capacity and gas exchange, leaf N concentration is correlated to the Gs, Tr, photosynthetic rate (Pn), and growth (Lei et al., 2012). A large part of N in plant tissues is present in protein, so protein concentrations are often directly related to total N concentrations (Bahrami et al., 2017).

Although some measures and mechanisms to improve plant nitrogen content under eCO_2_ have been reported on several occasions, such as protein extraction in the biorefinery (Solati et al., 2018), optimization the planting system (Manevski et al., 2017), improvement of photosynthesis and water use efficiency (Li, Wang & Liu, 2021; Manderscheid et al., 2018), increment of absorption and assimilation of ${\mathrm{NO}}_{3}^{-}$ (Bloom et al., 2014), as well as root formation and root elongation (Cohen et al., 2019), the combined effects of eCO_2_ and different N-fertigation levels on plant growth and N nutrition remain largely elusive. Particularly rare are studies measuring the effects of eCO_2_ and N on native grassland plant species in terms of productivity and N nutritional quality from root and leaf levels.

In the present study, we investigated the interactive effects of eCO_2_ plus N-fertilization on the root, shoot biomass, root length (RL), leaf area (LA), specific leaf area (SLA), leaf nitrogen concentration (LNC), Pn, Gs, Tr, and physiological processes associated with N acquisition of the dominant species of *Agropyron mongolicum*, a perennial rhizomatous C_3_ grass, in Ningxia desert steppe by growing them in soils collected from Ningxia desert steppe grassland using open-top chambers (OTCs). Our working hypothesis is that eCO_2_ and N-fertilization would further enhance biomass accumulation while increasing plant N nutrition under eCO_2_, where LNC, total root length (TRL), root N uptake capacity, root and leaf ${\mathrm{NO}}_{3}^{-}$ assimilation, as well as Gs and Tr, would play an important role in modulating the effects of the fertigation with N on plant growth and N nutritional quality under eCO_2_.

## Materials and Methods

### Plant materials and growth conditions

The experiment was conducted from April to August 2021 at the experimental farm of Ningxia University located in Yongning County, Ningxia, China (38°13′50″N; 106°14′21″E; 1116.76 m a. s. l.). Both seeds and soils were collected from the desert steppe of Yanchi County, Ningxia, China (37°20′30″N, 107°15′38″E). The soil was classified as sandy loam, having a pH of 8.55, soil organic carbon of 3.95 g kg^−1^, total N of 0.16 g kg^−1^, available phosphorus of 3.13 mg kg^−1^, and available potassium was 121.56 mg kg^−1^. The pots used were 15 L (27.5 cm in diameter at the top edge, 22 cm in diameter at the bottom, 31 cm in height). Before filling the pots, the soil was sieved passing through a two mm mesh. Thirty healthy seeds were sown in pots filled with 10 kg air-dried soil on 21st April 2021. All pots were well watered to ensure seedling establishment. After 1-month of growth outside the greenhouse, 8 uniform and robust seedlings of *A. mongolicum* were kept in each pot, and then all pots were moved into octagonal field open-top chambers (OTCs) on May 30, 2021, and CO_2_ and N treatments were initiated. Other environmental factors of all the OTCs (six chambers in total) were not significantly different among chambers and maintained with average temperature (28/18  ± 2 °C, day/night), relative humidity (70  ± 5%), soil moisture content (70–80% of field capacity), and light intensity (more than 500 µmol m^−2^ s^−1^ photosynthetic active radiation from natural sunlight). The actual CO_2_ concentrations, temperatures, relative humidity, and soil moisture content during the experimental period are shown in Fig. S1.

### Experimental design and treatments

This experiment was defined as a randomized split-plot design comprised of two CO_2_ levels (aCO_2_:400  ± 20 µmol mol^−1^ *vs* eCO_2_:800  ± 20 µmol mol^−1^) as the main plot and three N application rates (N0 (control): ambient; N1.2 (low N): ambient +1.2 g N m^−2^ yr^−1^ and N3.6 (high N): ambient +3.6 g N m^−2^ yr^−1^,) as the subplot. A total of 6 treatments, each treatment with 3 biological replicates. The eCO_2_ in OTCs was maintained by injecting pure CO_2_ and continuously monitored every 6 min using a system controller coupled to the proportional-integral controller. The automatic control system for CO_2_ levels and OTCs described in more detail by Ma et al. (2021). For N fertigation, all potted seedlings were moved into OTCs, randomly divided into three groups (15 pots per group) in each OTC, and 45 pots in total. Twice a month during the treatment period, each pot in OTCs received N fertilizer dissolved in 500 mL of tap water by spraying the seedlings in four equal splits at rates of total NH_4_NO_3_-N of 0, 1.2, and 3.6 g N m^−2^ yr^−1^ on 15th, 30th June, 15th, 30th July 2021, respectively. N application rates were designed based on our previous study (Xu et al., 2022). All pots were rotated every 7 days between the chambers to prevent the potential position effects and regularly weighed, watered, and maintained at 70–80% of field capacity until the end of the experiments. The plants were harvested for analysis after 70 days of the CO_2_ treatments.

### Determination of biomass and root morphology

Three whole individual plants were hand-harvested from per N treatment within each OTC at the harvested time. The harvested plants were separated into roots and shoots (leaves and stems) with scissors. The roots were washed with deionized water repeatedly, then each plant’s root and stem tissues were packed into separate envelopes and dried to constant weight at 65 °C. The dry weight of the shoots and roots of each plant was measured. The root/shoot ratio was calculated using the shoot and root dry weight ratio.

Fresh root samples from each treatment were analyzed for total root length (TRL) and surface area (RSA), the method of determination as described in a previous study by Xu et al. (2022). Specific root length (SRL) was calculated by dividing TRL by its mass (Cohen et al., 2019). RNU, as a surrogate for N uptake capacity per unit of root length, was calculated by the ratio of the total aboveground N content divided by TRL (Bahrami et al., 2017).

### Gas exchange and Leaf traits determinations

The leaf gas exchange assessments were performed on sunny days between 9:00–11:30 just before harvesting using an LI-6400 XT portable photosynthesis system (Li-Cor, Lincoln, NE, USA). Measurements were performed on the fully expanded leaves of each sample plant (at least three individual plants per treatment in each chamber) under continuous light photosynthetic photon flux density of 1,500 µmol m^−2^ s^−1^, 500 µmol s^−1^ flow rate, and air temperature of 25 °C. Three of the fully expanded leaves per replicate were excised from atop the stolons and were immediately scanned using an Epson flatbed scanner for calculation of total leaf area (LA) and then dried at 75 °C for 48 h until a constant dry weight (DW). The specific leaf area (SLA) was calculated as the ratio of leaf area to dry weight.

### Quantification of protein, amino acids, nitrate, and nitrogen concentration

Samples of 0.1 g were ground in liquid N and transferred to an Eppendorf^®^ tube (2.5 mL), and two mL of ultrapure water was added. The samples were centrifuged at 11, 200 × g for 10 min at 4 °C and the supernatant was collected. The quantification of ${\mathrm{NO}}_{3}^{-}$ was determined as described by Cataldo et al. (1975). Total protein content (TP) was measured using the Coomassie Brilliant Blue method (Bradford, 1976). The ninhydrin method was used to determine the total free amino acid content (FAA) (Yokoyama & Hiramatsu, 2003). The dried root and leaf samples were milled in a ball-mixer mill (MM200, Retsch, Haan, Germany) to analyze the nutrient content. The total N concentration was measured using an elemental analyzer (Flash EA1112; Thermo Scientific, West Palm Beach, FL, USA). Total N content was calculated as the tissue N concentration × tissue total dry weight. The percentage of N remaining as unassimilated ${\mathrm{NO}}_{3}^{-}$ in the organs was calculated as ${\mathrm{NO}}_{3}^{-}$ -N content/total N content (Bahrami et al., 2017).

### Nitrate reductase and glutamine synthetase activity

Nitrate reductase (NR, E.C.1.6.6.1) and glutamine synthetase (GS, EC 6.3.1.2) determinations were made according to Domiciano et al. (2020). The enzymatic extracts were obtained by homogenizing 1.0 g of frozen tissue and adding 50mM potassium phosphate buffer (pH 7.5) containing 1 mM phenylmethanesulfonyl fluoride, 5 mM ethylene diamine tetra-acetic acid (EDTA), 10% polyvinylpyrrolidone and 2 mM DL-dithiothreitol. The extract was centrifuged at 13,000 × g for 20 min at 4 °C, and the supernatant was used as the source of the enzymes (Domiciano et al., 2020). The NR activity was measured according to the protocol method (Berges & Harrison, 1995). GS was determined using the protocol of Ratajczak, Ratajczak & Mazurowa (1981).

### Statistical analysis

Two-way analyses of variance (ANOVAs) were used to examine the effects of CO_2_, N-fertigation, and the interaction between CO_2_ concentration and N-fertilization on the morphological and physiological traits in *A. mongolicum*. Also, one-way analysis of variance (ANOVA) with Duncan’s range test to compare the difference between the aCO_2_ and eCO_2_ combined with different N level treatments on morphological and physiological traits at a 0.05 probability level. All these tests were manipulated using SPSS 21.0 software (SPSS Inc., Chicago, IL, USA). Redundancy analysis (RDA) with a forward selection procedure was used to determine morphological and physiological parameters significantly related to the growth and N nutrient content of *A. mongolicum*. Prior to analysis, variables that had high variance inflation factors (VIF > 20) were removed from the model to eliminate multicollinearity. We further analyze the relationships of the growth and N nutrient content with morphophysiological and biochemical traits using the “GeneNT” package in R software (Asif et al., 2020), and the network visualization was conducted using Cytoscape software (version 3.9.9) (Shannon et al., 2003).

## Results

### Biomass, root, and leaf morphology traits

**Figure 1:**
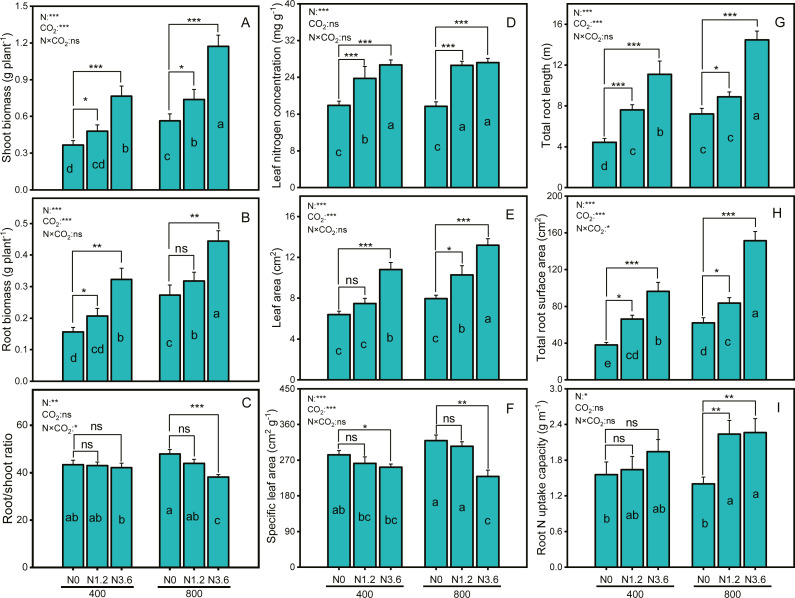
Differences in biomass, root/shoot, root, and leaf morphology variables of *A. mongolicum* under ambient (400 ± 20 ppm) and elevated CO_2_ concentrations (800 ± 20 ppm) combined with control (N0), low (N1.2), and high (N3.6) nitrogen levels. Results of two-way ANOVAs for variables and the interaction of factors are shown in the figure, asterisks (*, **, ***) and ns denote significances at *p* < 0.05, *p* < 0.01, *p* < 0.001, and no significance, respectively. Different lowercase letters in vertical bars indicate significant differences among the treatments by Duncan’s test at *p* < 0.05. Asterisks (*, **, ***) and ns above vertical bars indicate significant difference for comparison between N0 and N1.2, N0 and N3.6 treatments in a CO_2_ and eCO_2_ at *p* < 0.05, *p* < 0.01, *p* < 0.001, and no significance, respectively, analyzed by Student’s test. Values are means ± SE (*n* = 9).

Biomass of both shoot and root in *A. mongolicum* exhibited consistently faster growth in all N treatments compared to N0 under both CO_2_ conditions (Figs. 1A and 1B). Compared with aCO_2_, eCO_2_ exerted greatly growth stimulation under different N treatments, with a 30.8% increase only in the shoot biomass under low N, whereas 39.9% and 107.6% stimulation under high N in root and shoot biomass, respectively. The different responses of the shoot and root biomass in *A. mongolicum* to enriched CO_2_ andN led to a decline in the root/shoot ratio. The root/shoot ratios of *A. mongolicum* were 43.4%, 43%, and 42.2% under aCO_2_ at control, low, and high N treatments, remaining relatively constant changes; whereas under eCO_2_, the ratios were increased to 47.9%, 43.9%, and 38.1%, respectively. The results of two-way ANOVA indicated that CO_2_ concentration and N-fertilization level had significant effects on the shoot and root biomass, but their interaction had no significant effects. However, changes in root/shoot ratio were depending on N and interactions between CO_2_ concentration and N fertilization, while CO_2_ concentration had no impact on the root/shoot ratio.

Leaf nitrogen concentration (LNC) and leaf area (LA) of *A. mongolicum* were significantly increased under both CO_2_ conditions at low and high N treatments (Figs. 1D and 1E), while the same treatments caused a significant reduction in SLA of *A. mongolicum* (Fig. 1F). High N supplement increased LNC and LA in *A. mongolicum* by 49.3% and 69% at aCO_2_, and by 53.9% and 65.7% at eCO_2_, while decreased 11.1% and 28.2% in the SLA under both CO_2_ conditions, respectively, compared to control treatment. Moreover, both CO_2_ concentration and N addition level had significant effects on the LA and SLA, but LNC was only affected by N-fertigation, the interaction between CO_2_ concentration and N levels had no significant effect on any one of these variables.

N-fertilization stimulated the root morphology of *A. mongolicum* under both aCO_2_ and eCO_2_, for TRL, a 150.1% and 100.4% increase at high N treatment (Fig. 1G), whereas153.9% and 144.7% stimulation in RSA with high N treatment at aCO_2_ and eCO_2_ (Fig. 1H), respectively. There was no significant difference in root N uptake capacity under aCO_2_, while it’s significantly increased with increasing N supplement under eCO_2_, with a 61.6% increase at high N level (Fig. 1I). Furthermore, both CO_2_ concentration and N-fertilization level had significant effects on the TRL and RSA, their interaction had only effect on RSA, while root N uptake capacity was only affected by N-fertigation.

### Leaf gas exchange

**Figure 2:**
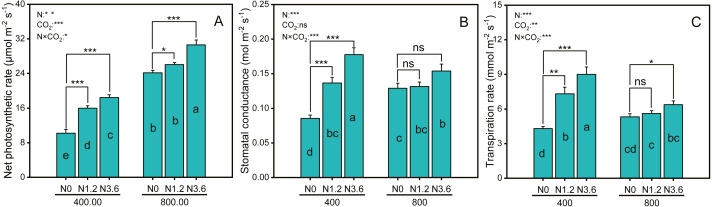
Differences in gas exchange parameters of *A. mongolicum* under ambient (400 ± 20 ppm) and elevated (800 ± 20 ppm) CO_2_ concentrations combined with control (N0), low (N1.2), and high (N3.6) nitrogen levels. Results of two-way ANOVAs for variables and the interaction of factors are shown in the text, asterisks (*, **, ***) and ns denote significances at *p* < 0.05, *p* < 0.01, *p* < 0.001, and no significance, respectively. Different lowercase letters in vertical bars indicate significant differences among the treatments by Duncan’s test at *p* < 0.05. Asterisks (*, **, ***) and ns above vertical bars indicate significant difference for comparison between N0 and N1.2, N0 and N3.6 treatments in ambient CO_2_ and elevated CO_2_ at *p* < 0.05, *p* < 0.01, *p* < 0.001, and no significance, respectively, analyzed by Student’s test. Values are means ± SE (*n* = 9).

Net photosynthetic rate (Pn) and transpiration rate (Tr) were significantly affected by CO_2_, N-fertigation, and the interaction between CO_2_ concentration and N-fertilization (Figs. 2A and 2C), while stomatal conductance (Gs) was significantly affected by N-fertilization and their interaction (Fig. 2B). The Pn of *A. mongolicum* increased by 80.8% and 26.9%, respectively, at high N treatment compared to that at the control treatment under both aCO_2_ and eCO_2_. N-fertigation promoted the increase of the Gs and Tr under ambient and elevated CO_2_, although not statistically significant in eCO_2_ condition. High N-fertigation increased the Gs and Tr of *A. mongolicum* by 107.4% and 109% at aCO_2_, respectively, compared to control treatments.

### Total N, amino acid, protein, NR, and GS activity in root and leaf

**Figure 3:**
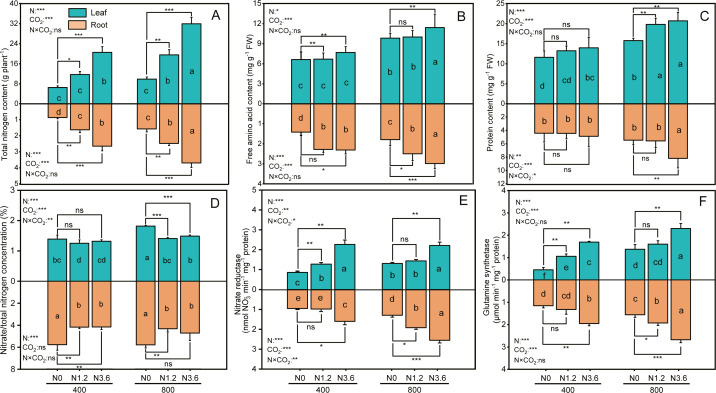
The effect of ambient (400 ± 20 ppm) and elevated (800 ± 20 ppm) CO_2_ concentrations combined with control (N0), low (N1.2), and high (N3.6) nitrogen levels on physiological traits in leaf and root of *A. mongolicum*. Results of two-way ANOVAs for variables and the interaction of factors are shown in the text, asterisks (*, **, ***) and ns denote significances at *p* < 0.05, *p* < 0.01, *p* < 0.001, and no significance, respectively. Different lowercase letters in vertical bars indicate significant differences among the treatments by Duncan’s test at *p* < 0.05. Asterisks (*, **, ***) and ns above vertical bars indicate significant difference for comparison between N0 and N1.2, N0 and N3.6 treatments in ambient CO_2_ and elevated CO_2_ at *p* < 0.05, *p* < 0.01, *p* < 0.001, and no significance, respectively, analyzed by Student’s test. Values are means ± SE (*n* = 3).

The content of total nitrogen (TN), free amino acid, and protein in root and shoot was significantly affected by CO_2_ concentration and N-fertilization (Figs. 3A, 3B and 3C), the eCO_2_ increased TN of root and shoot by 50.6% and 58.3%, respectively, compared to aCO_2_. The TN of root and shoot grown under high N at aCO_2_ conditions was increased by 205.5% and 213.9%, respectively, but by 134.4% and 223.1% at eCO_2_, compared to that under control treatment. However, the interaction between N and CO_2_ did not have significant influences on these variables in both root and shoot, except for protein content in the root. When comparing the effect of N-fertigation on the free amino acid content, the increase observed in the root and leaf were 62.9% and 16.1% at aCO_2_ and 66.5% and 16.1% at eCO_2_ under high N treatment, respectively, compared to control treatments. The content of protein in leaf and root progressively increased from control to high N treatment, with high N treatment increasing protein content in leaf by 20.3% and 31.3% under both a CO_2_ and eCO_2_, respectively, compared to control treatment, and this increase was higher for eCO_2_ than aCO_2_ plants.

The ratio of nitrate to total nitrogen (${\mathrm{NO}}_{3}^{-}$/TN) in leaf decreased by 5.5% and 21.9% at high N under eCO_2_ and aCO_2_, respectively, compared to control treatment, while decreased 38.5% and 22.6% in root under both CO_2_ conditions. However, this decrease did not differ among N treatments under aCO_2_ (Fig. 3D). The NR in leaf and root was significantly increased with increasing N addition under both CO_2_ conditions. Compared to control treatment, high N treatment increased NR in leaf by 162.6% and 69.4% and by 67.8% and 96.7% in root under both a CO_2_ andeCO_2_ (Fig. 3E), respectively. There was an increase in GS activity in leaf and root for all the N treatments when exposure to both CO_2_ conditions, GS activity in leaf and root treated with high N treatment was 67.8% and 71.9% at eCO_2_, respectively, higher than that of control treatment and same N treatments at aCO_2_ conditions (Fig. 3F). In addition, except for ${\mathrm{NO}}_{3}^{-}$/TN in root only affected by N-fertilization, the ${\mathrm{NO}}_{3}^{-}$/TN, GS, and NR activity, across all leaf and root, was significantly affected by CO_2_ and N-fertigation, respectively, while the interaction between N and CO_2_ only influenced the ${\mathrm{NO}}_{3}^{-}$/TN of leaf and the NR in leaf and root.

### Multivariate analysis of traits for growth and N nutrient of *A. mongolicum*

**Figure 4:**
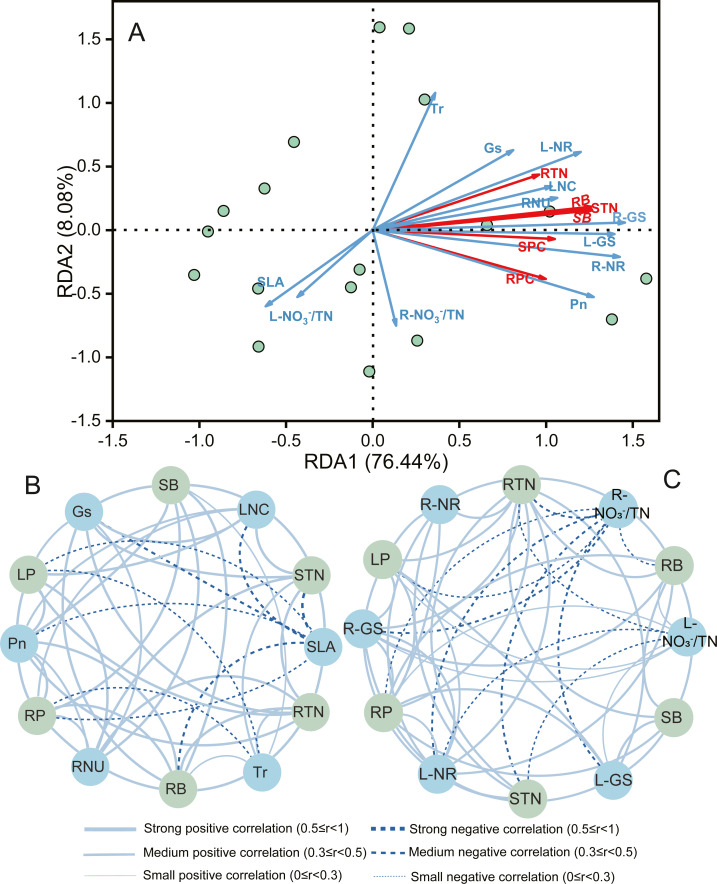
Redundancy analysis (RDA) and correlation network analysis to determine the relationships among morphophysiological and biochemical traits with biomass, total N content, and protein content of *A. mongolicum*. SLA, specific leaf area; LA, leaf area; LNC, leaf nitrogen content; Pn, net photosynthetic rate; Gs, stomatal conductance; Tr, transpiration rate; RL, root length; RSA, root surface area; RNU, N uptake capacity per unit of root length; L- ${\mathrm{NO}}_{3}^{-}$/TN, leaf unassimilated ${\mathrm{NO}}_{3}^{-}$; R- ${\mathrm{NO}}_{3}^{-}$/TN, root unassimilated ${\mathrm{NO}}_{3}^{-}$; L-NR, leaf nitrate reductase; R-NR, root nitrate reductase; L-GS, leaf glutamine synthetase; R-GS, root glutamine synthetase; RTN, root total nitrogen content; STN, shoot total nitrogen content; SB, shoot biomass; RB, root biomass; SPC, shoot protein content; RPC, root protein content.

Constrained redundancy analysis (RDA) performed for *A. mongolicum* indicated that the first two axes explained 84.5% of the total variance (Fig. 4A). Root and shoot biomass showed positive correlations with LNC, RNU, Pn, RTN, STN, SPC, and RPC were positively related to LNC, Gs, Tr, RNU, L-GS, R-GS R-NR, L-NR; whereas, except for the above, RPC and RTN showed negative correlations with L- ${\mathrm{NO}}_{3}^{-}$/TN and R- ${\mathrm{NO}}_{3}^{-}$/TN, respectively (Table S1). These findings indicated that the growth and N nutrient content of *A. mongolicum* were principally influenced by root and leaf morphophysiological as well as biochemical characteristics under eCO_2_ and N-fertilization.

To further reveal the correlations between the various traits and both growth and N nutrient, correlation networks were constructed (Figs. 4B and 4C). The results also showed that L-GS, Gs, Pn, and RNU was strongly and positively correlated with the shoot and root biomass (*r* > 0.5). Furthermore, the STN was strongly and positively correlated with the LNC, RNU, Tr, Gs, R-NR, L-NR, R-GS, and GS (*r* > 0.5); the RTN was strongly and positively correlated with the RNU, Tr, Gs, R-NR, L-NR, R-GS, L-GS, and GS (*r* > 0.5); while they were mediumly and negatively correlated with L- ${\mathrm{NO}}_{3}^{-}$/TN and R- ${\mathrm{NO}}_{3}^{-}$/TN, respectively (0.3 ≤ *r* < 0.5). Similar results have also been found in total protein content, the SPC and RPC were a strongly and mediumly positive correlation with the LNC, RNU, R-NR, R-NR, R-GS, and L-GS (*r* ≥ 0.3), and they were a small negative correlation with SLA and R- ${\mathrm{NO}}_{3}^{-}$/TN ( *r* < 0.3), respectively.

## Discussion

This study clearly showed that the increase in atmospheric CO_2_ played a major role in *A. mongolicum* growth and N nutrition and that this role is determined by the available N. This interaction demonstrated that the responses of plants to the eCO_2_ and N-fertilization are dependent on the coordination between root and leaf development and are associated with the metabolic implications of the available N. Our study showed that although both CO_2_ conditions increased root and shoot biomass, eCO_2_ combined with the increased N supplement accelerated the effect of the eCO_2_ on the growth stimulation of plants, demonstrating that the promotion of *A. mongolicum* growth under the short-term enrichment of air CO_2_ is dependent on the available N (Figs. 1A and 1B). A previous study has shown that plant maximum growth response to eCO_2_ could be improved by the effects of eCO_2_ on the leaf carboxylation rate per unit leaf area or relative growth rate when plants received adequate N for growth (Leakey et al., 2009; Liu et al., 2012). Our growth stimulation can be partly explained by the increment in leaf photosynthesis that occurs when *A. mongolicum* is grown under eCO_2_ plus N-fertilization. The SLA expressed as the leaf area per unit of dry weight is one of the most widely used traits for describing leaf characteristics and their relationship with photosynthesis (Burgess & Huang, 2014). In our study, the combination of higher leaf N content and a lower SLA indicated that *A. mongolicum* developed under eCO_2_ plus N-fertilization conditions had a smaller leaf area per unit biomass or the leaves become thicker, which was consistent with some studies (Burgess & Huang, 2014; Lei et al., 2012; Liu et al., 2012). It was shown that tree species grown in elevated CO_2_ combined with increased N-fertilization had thicker leaves and a greater leaf weight per unit leaf area (Liu et al., 2012). It was suggested that the thicker leaves with increased mesophyll and cell N content of leaves may be a reason for the observed increase in photosynthetic rates (Jauregui et al., 2016). Our study confirmed the conclusion that leaf area per unit biomass decreased and the photosynthetic rate increased upon exposure to 800 µmol mol^−1^ CO_2_ (Figs. 1F and 2A). Furthermore, leaf thickness has also been shown to have a close relationship with the transpiration rate wherein thicker leaves have greater transpiration efficiency, which was to be beneficial to the nutritional uptake of plants (Giuliani et al., 2013), increased N content in the leaves in eCO_2_ could also alleviate photosynthetic acclimation to CO_2_ enrichment, enhancing photosynthetic rates of leaves (Vicente et al., 2015).

The increased shoot biomass in *A. mongolicum* was mainly due to enhanced leaf photosynthesis, as discussed above. Likewise, roots, such as root morphology and architecture, also played an important role in plant growth responses to eCO_2_ (Burgess & Huang, 2014). A review described the effects of eCO_2_ on plant root systems and reported that both the root number and root length are significantly increased due to eCO_2_ and other interacting factors, such as warming, precipitation, and nutrient availability (Leakey et al., 2009). In a comprehensive review, it was also reported that other structural aspects of root growth tend to increase when plants are maintained at eCO_2_ levels, including the volume, branching, and relative growth rate, whereas reports of changes to the root N uptake capacity due to eCO_2_ level are lacking (Rogers, Runion & Krupa, 1994). As expected, root length, root surface area, and N uptake capacity of *A. mongolicum* grown under at eCO_2_ various N conditions were significantly increased compared to that at the same N treatment under aCO_2_ (Figs. 1G and 1H), and the degree of increase relative to ambient controls was highly dependent upon the N availability. The stimulation of root growth can have a substantial effect on plant growth due to greater soil volume exploration in deeper soil strata. Most interestingly, with the increase in N treatments, the response of the root/shoot ratio of *A. mongolicum* to changes in CO_2_ concentrations was contrary to the finding of studies with increases in R/S previously reported (Cohen et al., 2019). However, this is less surprising when we considered the available resources since increased root growth requires both C and N inputs. It has been reported that plants with adaptation to low N treatment often allocate relatively more biomass to roots and enhanced root nutrients uptake capacity, consequently, eCO_2_ enhances root growth more than shoot growth(Lei et al., 2012; Liu, Tian & Zhang, 2016). With the high N treatment, however, the increase in available C due to increased photosynthesis, coupled with a non-limiting N supply, made it possible to increase the shoot growth substantially (Vicente et al., 2015). Root proliferation through increased length or surface area serves as a critical adjustment function for water or nutrients uptake and adaptation mechanisms to an increased rate of carbon and nitrogen cycling have been implicated in enhancing plant productivity or nutritional quality in an agro-ecosystem (BassiriRad, Gutschick & Lussenhop, 2001; Leakey et al., 2009; Liu et al., 2012).

It is well known that eCO_2_ induces stomatal closure can decline Gs and Tr of plants while decreasing the transpiration-driven mass flow of N, consequently decreasing N uptake by roots (Jayawardena et al., 2021; Li, Wang & Liu, 2021; McGrath & Lobell, 2013). Consistent with this, the results from the present study showed that eCO_2_ significantly decreased Gs and Tr compared with aCO_2_ (Figs. 2B and 2C), however, high N supply increased Gs and Tr to approximately 1.5 and 1.6 times that of control N treatments, respectively, regardless of the CO_2_ treatments. Accumulated evidence has revealed that increasing the nutrient supply from low to high can increase plant N uptake by increasing Gs as well as Tr of plants (De Oliveira et al., 2012; Lotfiomran, Kohl & Fromm, 2016), consistent with the results from the present study. Therefore, we suggest that the increase of both stomatal Gs and Tr by adding N under eCO_2_ could weaken the negative effect of eCO_2_ in decreasing the transpiration-driven mass flow of N.

The protein decrease under eCO_2_ was not only related to the external growth development limitations to N uptake but also internal signals arising from the progress imposed by physiological metabolism, most prominently inhibition of ${\mathrm{NO}}_{3}^{-}$ assimilation (Bloom et al., 2014). The ${\mathrm{NO}}_{3}^{-}$/total N ratio was used as a surrogate for plant unassimilated ${\mathrm{NO}}_{3}^{-}$ and negatively correlated with total protein in plant tissues (Bahrami et al., 2017; Jayawardena et al., 2021). The present study showed that increasing N-fertilization did not influence the leaf ${\mathrm{NO}}_{3}^{-}$ to total N ratio at aCO_2_, but it decreased the leaf ${\mathrm{NO}}_{3}^{-}$ to total N ratio at eCO_2_ and root ${\mathrm{NO}}_{3}^{-}$ to total N ratio under both CO_2_ conditions (Fig. 3D), and shoot and root protein content negatively correlated with L- ${\mathrm{NO}}_{3}^{-}$/TN and R- ${\mathrm{NO}}_{3}^{-}$/TN, respectively (Figs. 4A and 4B), indicating N-fertilization could alleviate the effects of eCO_2_ on inhibition of ${\mathrm{NO}}_{3}^{-}$ photo-assimilation of *A. mongolicum*. The proportion of unassimilated ${\mathrm{NO}}_{3}^{-}$ was declined, either by assimilation of ${\mathrm{NO}}_{3}^{-}$ or by an increase in total N, or both (Bahrami et al., 2017). With increased N supply, the total N content and the free amino acids in leaves and roots of *A. mongolicum* significantly increased when plants were exposed to two CO_2_ conditions (Figs. 3A and 3B), suggesting the above both occurred. Supporting the possibility of enhancement of ${\mathrm{NO}}_{3}^{-}$ assimilation, we saw an increase in the concentrations of N-assimilatory proteins, such as NR and GS activities (Figs. 3E and 3F), at eCO_2_ and N-fertilization, and positively correlated with LPC and RPC (Fig. 4B), which is in agreement with previous studies (Bahrami et al., 2017; Jauregui et al., 2016; Jayawardena et al., 2017). Croy & Hageman (1970), who used increased NR activity as a proxy for a decline of unassimilated ${\mathrm{NO}}_{3}^{-}$, explained a positive correlation between leaf NR activity and grain protein in wheat. Notably, the NR and GS activity in leaves and roots were able to maintain relatively similar levels among all same treatment combinations; therefore, we inferred that the proportion of ${\mathrm{NO}}_{3}^{-}$ in total N in roots which appeared to be more highly than in leaves might be mainly because of the large increase in total N content in leaves. Furthermore, as previous studies discussed, the positive effect of eCO_2_ on plants growth during different N sources was significantly correlated with ${\mathrm{NO}}_{3}^{-}$ uptake and assimilation (Domiciano et al., 2020; Jayawardena et al., 2017). Taken together, our results suggest that N-fertilization could alleviate inhibition of ${\mathrm{NO}}_{3}^{-}$ assimilation under eCO_2_ by increasing concentrations of N-assimilatory proteins and enhancing ${\mathrm{NO}}_{3}^{-}$ assimilation, consequently, improving productivity and quality of *A. mongolicum*.

## Conclusions

N-fertigation causes photosynthesis improvement *via* increased leaf N content and reduced specific leaf area, resulting in enhanced stimulation for growth by eCO_2_, but this stimulation does not affect the N nutrition of *A. mongolicum* under eCO_2_. Under sufficient N supply, the reduced proportion of ${\mathrm{NO}}_{3}^{-}$ in total leaf and root N, increased leaf N content, root length, and Tr are not only associated with N concentrations in vegetative plant parts, but also with the total protein content of *A. mongolicum* grown at eCO_2_. Therefore, with the application of effective management measures of N-fertilization, grass productivity and quality may enhance under the CO_2_ levels anticipated during the end of this century.

##  Supplemental Information

10.7717/peerj.14273/supp-1 Supplemental Information 1 Supplemental Figure and Table

10.7717/peerj.14273/supp-2 Supplemental Information 2 Raw data

## Additional Information and Declarations

### Data Availability

The following information was supplied regarding data availability: The raw measurements are available in the Supplemental File.
