# Supplementary material for: Nitrogen fertilization and CO2 concentration synergistically affect the growth and protein content of Agropyron mongolicum
Source: PeerJ. 2022 Oct 31;10:e14273. doi: 10.7717/peerj.14273 (PMC9632468; doi:10.7717/peerj.14273)
Supplement: Supplemental Information 1 [file peerj-10-14273-s001.docx]

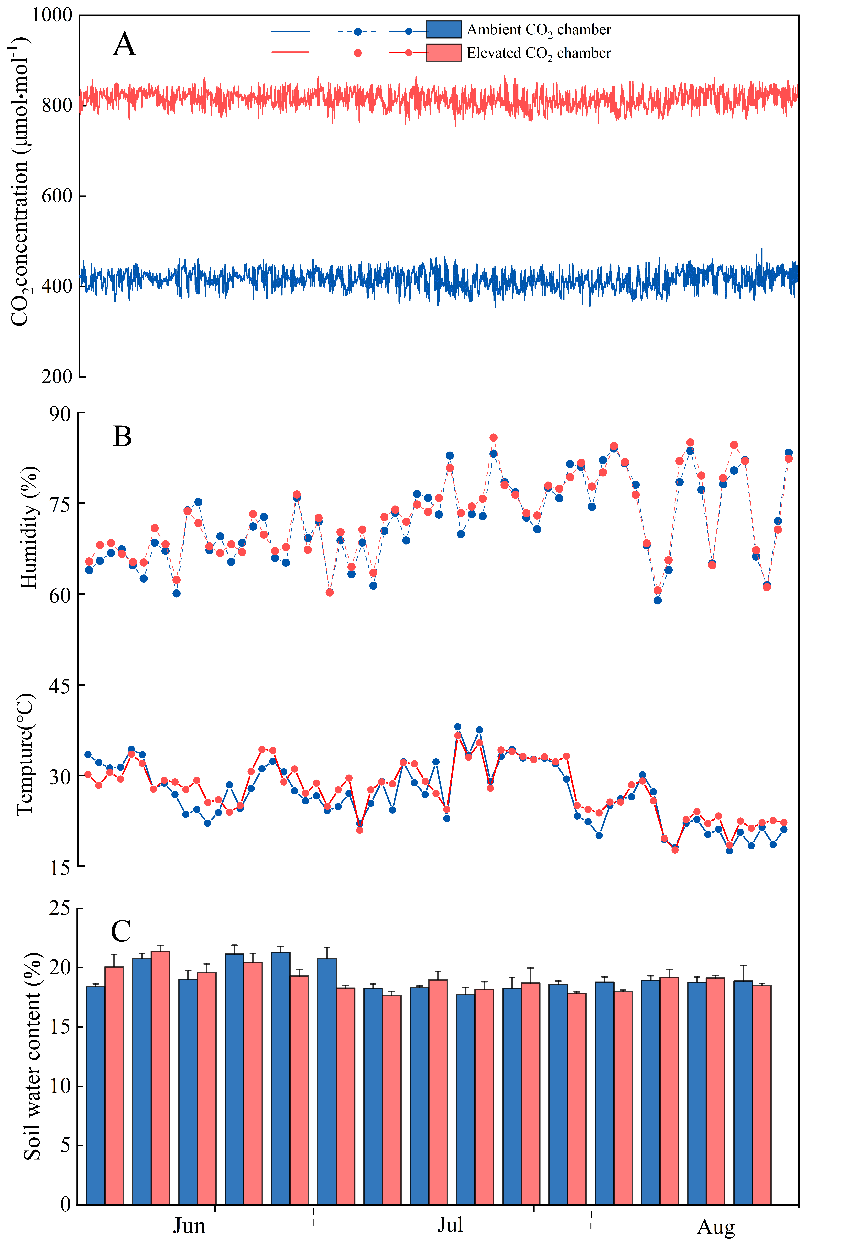


**Figure S1.** The actual CO_2_ concentrations (a), relative humidity (b), temperatures (c), and soil moisture content (c) exposure to ambient (400 ± 20 ppm) and elevated CO_2_ (800 ± 20 ppm) during the experimental period.

**Table S1** Results by redundancy analysis (RDA) ordination with the first two axes and Monte Carlo permutation test

| Explanatory variables | RDA1 | RDA2 | R^2^ | *P* |
| --- | --- | --- | --- | --- |
| SLA | -0.735 | -0.677 | 0.321 | 0.050 * |
| LNC | 0.948 | 0.317 | 0.521 | 0.002 * |
| Pn | 0.937 | -0.347 | 0.812 | 0.001*** |
| Gs | 0.801 | 0.598 | 0.457 | 0.006** |
| Tr | 0.344 | 0.938 | 0.5462 | 0.004** |
| RNU | 0.972 | 0.233 | 0.530 | 0.005** |
| L-NO_3_^-^/TN | 0.175 | -0.984 | 0.237 | 0.191 |
| R-NO_3_^-^/TN | -0.661 | -0.750 | 0.200 | 0.001*** |
| R-NR | 0.992 | -0.123 | 0.897 | 0.001*** |
| L-NR | 0.892 | 0.450 | 0.795 | 0.001*** |
| R-GS | 0.998 | 0.053 | 0.931 | 0.001** |
| L-GS | 0.999 | -0.007 | 0.851 | 0.001*** |

SLA, specific leaf area; LNC, leaf nitrogen content; Pn, net photosynthetic rate; Gs, stomatal conductance; Tr, transpiration rate; RNU, N uptake capacity per unit of root length; L-NO_3_^-^/TN, leaf unassimilated NO_3_^-^; R-NO_3_^-^/TN, root unassimilated NO_3_^-^; L-NR, leaf nitrate reductase; R-NR, root nitrate reductase; L-GS, leaf glutamine synthetase; R-GS, root glutamine synthetase. **p* ≤ 0.05, ***p* ≤ 0.01, ****p* ≤0.001.
